# Extract, transform, load framework for the conversion of health databases to OMOP

**DOI:** 10.1371/journal.pone.0266911

**Published:** 2022-04-11

**Authors:** Juan C. Quiroz, Tim Chard, Zhisheng Sa, Angus Ritchie, Louisa Jorm, Blanca Gallego

**Affiliations:** 1 Centre for Big Data Research in Health, UNSW, Sydney, Australia; 2 Concord Clinical School, University of Sydney, Sydney, Australia; 3 Health Informatics Unit, Sydney Local Health District, Camperdown, Australia; University of Braunschweig - Institute of Technology, GERMANY

## Abstract

Common data models standardize the structures and semantics of health datasets, enabling reproducibility and large-scale studies that leverage the data from multiple locations and settings. The Observational Medical Outcomes Partnership Common Data Model (OMOP CDM) is one of the leading common data models. While there is a strong incentive to convert datasets to OMOP, the conversion is time and resource-intensive, leaving the research community in need of tools for mapping data to OMOP. We propose an extract, transform, load (ETL) framework that is metadata-driven and generic across source datasets. The ETL framework uses a new data manipulation language (DML) that organizes SQL snippets in YAML. Our framework includes a compiler that converts YAML files with mapping logic into an ETL script. Access to the ETL framework is available via a web application, allowing users to upload and edit YAML files via web editor and obtain an ETL SQL script for use in development environments. The structure of the DML maximizes readability, refactoring, and maintainability, while minimizing technical debt and standardizing the writing of ETL operations for mapping to OMOP. Our framework also supports transparency of the mapping process and reuse by different institutions.

## Introduction

Electronic health records (EHRs), administrative data, clinical registries, and linked data enable observational studies and evidence-based research that leverage the data of large and heterogeneous populations [1–3]. The growing availability of EHR-linked biobanks also facilitates research and implementation of patient phenotyping and personalized medicine [4]. Depending on the location, context, and purpose of the data, different datasets store information using different structures and semantics, which makes conducting analysis across them a challenge. Common data models offer a solution by standardizing the structure and semantics of data. The Observational Medical Outcomes Partnership Common Data Model (OMOP CDM), managed by the Observational Health Data Sciences and Informatics (OHDSI), continues to be one of the leading common data models for leveraging clinical and administrative health data for research purposes [5]. Since its introduction, different types of health databases have been mapped to OMOP CDM: EHRs [6–10], claims datasets [9, 11, 12], biospecimen data [13], and registries [14].

Converting routinely collected health data to OMOP is driven by the aims of: (1) efficiency–reuse of analytics tools and software, (2) transparency and reproducibility—compare findings from different data sources with a variety of methods without sharing the actual data and protecting patient privacy, and (3) scalability—conducting studies by leveraging the data from multiple locations and settings [5, 15]. The need for OMOP also stems from health databases originating or being designed to track patients within a hospital, mainly for administrative purposes such as billing and managing claims, not for conducting observational studies or other study designs [2, 16]. At its best, data in OMOP allows for multicenter observational studies, allowing for models to be externally validated across health datasets over the world [5, 17, 18].

Converting a dataset to the OMOP CDM entails the development of an extract, transform, load process (ETL), which converts both the structure and semantics of the original data to the standards defined by the OMOP CDM. Conceptually, the mapping process identifies how fields in the source health datasets relate to the fields in OMOP CDM and the data transformations that need to take place. Resources currently available to a team or institution interested in converting their data to OMOP include: OMOP experts, other users who have mapped data to OMOP, OHDSI web forums, OHDSI tools, and private companies that perform the ETL process for a fee.

In contrast to generic ETL tools (i.e. Talend Open Studio), the OHDSI tools (i.e. White Rabbit, Rabbit in a Hat) were developed exclusively for mapping data to OMOP. White Rabbit helps explore and understand the source database, while Rabbit in a Hat visually documents the mapping from source to OMOP [19]. However, the graphical approach of Rabbit in a Hat and ETL tools such as Talend Open Studio becomes visually cumbersome when dealing with a large number of tables and columns. Confusing visual clutter is compounded when dealing with complex mapping logic involving multiple source tables—commonly encountered in the mapping of sophisticated, proprietary relational databases used by commercial EHRs. Tools are required that improve the ETL process, complement existing generic and OHDSI ETL tools, enable mapping efforts from research institutions to be shared, and standardize the writing of mapping operations for complex and simple datasets.

The aim of this paper is to develop an ETL framework for the conversion of health databases to the Observational Medical Outcomes Partnership Common Data Model (OMOP CDM) that supports transparency of the mapping process, readability, refactoring, and maintainability. Our proposed ETL framework uses a data manipulation language (DML) that organizes SQL in YAML, a widely used human-readable serialization language. The ETL framework was developed as part of our ongoing work mapping Cerner Millennium (Cerner Corporation, Kansas City, MO, USA, “CERNER”) electronic health records used by Australian Local Health Districts to OMOP CDM, and all examples presented in this paper use this CERNER to OMOP conversion to showcase the DML.

### Extract, transform, load framework

**Fig 1:**
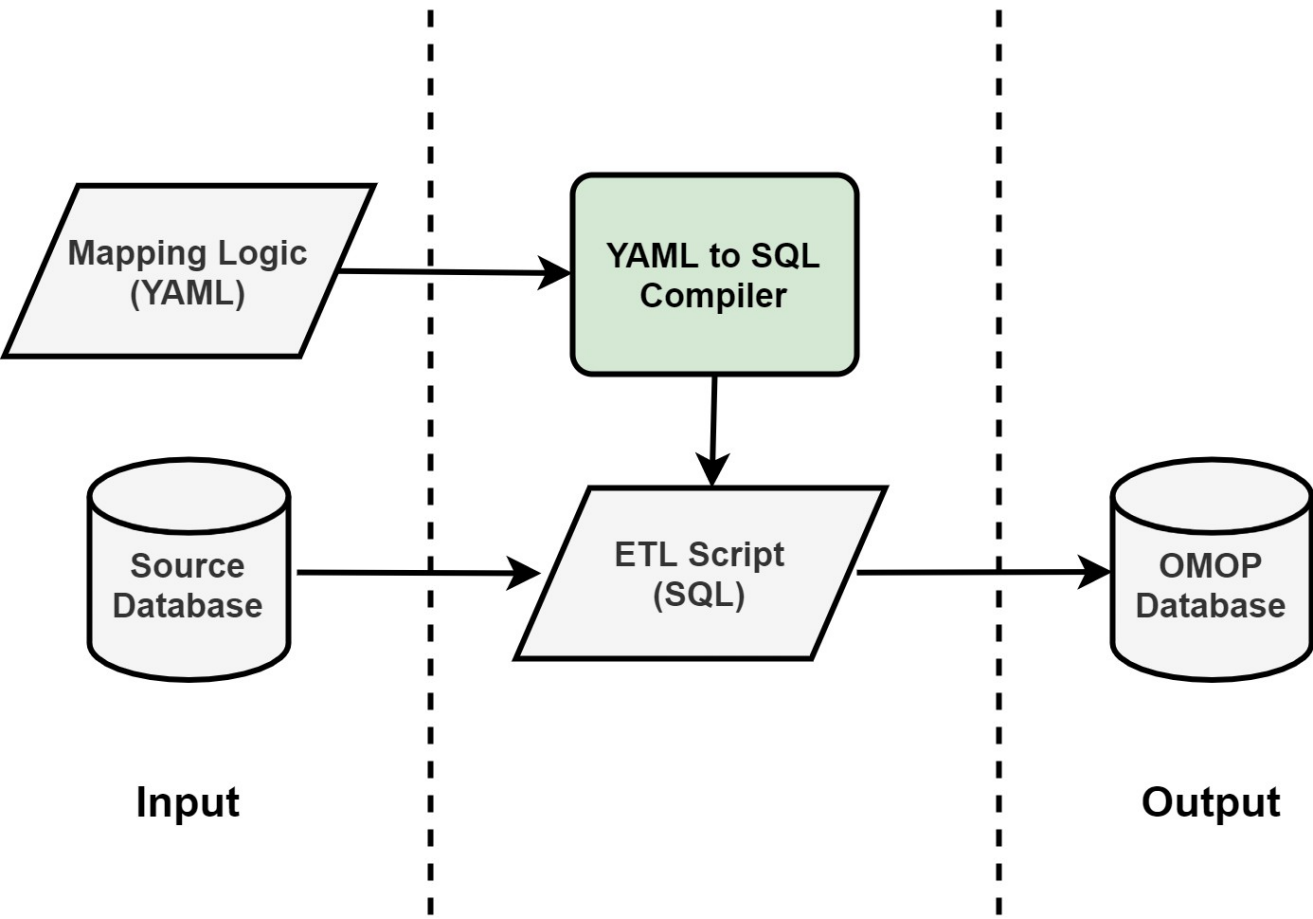
Architecture of the extract, transform, load (ETL) framework. A compiler converts mapping logic, written by organizing SQL snippets in YAML key-value pairs, to an ETL SQL script, which contains all the executable operations to map from the source database to OMOP.

**Fig 2:**
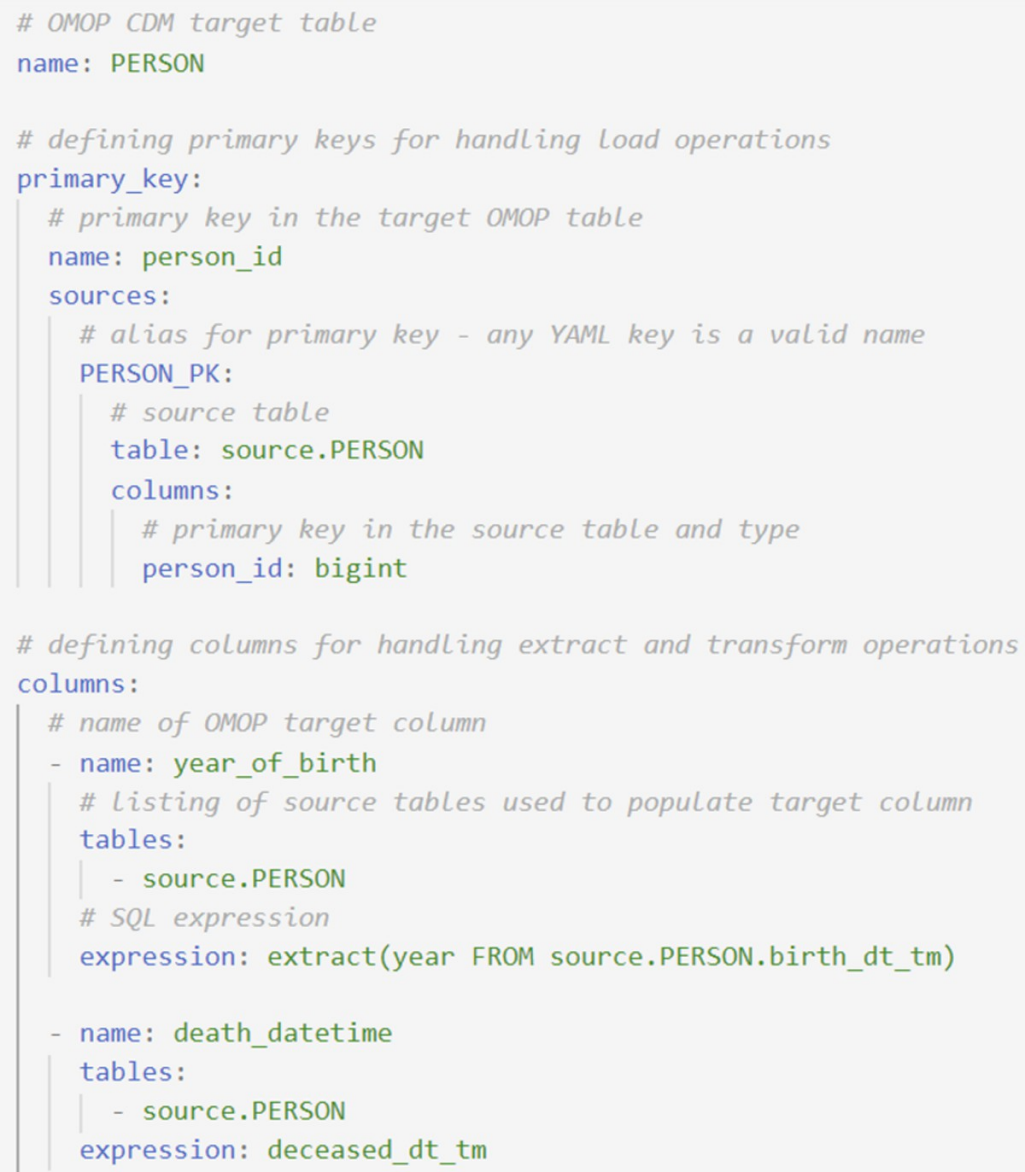
Source to OMOP YAML file structure. Rules for mapping from the CERNER PERSON table to the OMOP PERSON table, with rules defined for two columns of the OMOP PERSON table: year_of_birth and death_datetime. For each target table, the mapping rules are defined on a column-by-column basis using SQL snippets organized into YAML fields. Mapping a table requires three sections: (1) name of the OMOP table being mapped, (2) definition of primary keys used by the ETL framework to manage the load (insert) operations, and (3) mapping rules for columns in the OMOP table.

Fig 1 illustrates the architecture of our ETL process from a source database to the target OMOP CDM dataset. A compiler reads rules written in our data manipulation language (DML) and generates an ETL SQL script containing all the executable operations to extract, transform, and load the data from the source database to OMOP. The ETL script can then be used in any development environment. Access to the source code of the compiler is available at https://github.com/clinical-ai/omop-etl. See S1 File for an example of the SQL script generated from the YAML in Fig 2.

### Data manipulation language

The DML uses YAML and PostgreSQL syntax. YAML is a human-readable data format, commonly used for storing configuration settings and metadata of software programs. The DML uses YAML key-value pairs to define the source data, the target OMOP tables and columns, and the extract, transform, and load operations to map from source data to OMOP. Statements for extraction from the source schema are coded in the YAML values.

The source-to-OMOP ETL operations are organized by OMOP table (i.e. PERSON, OBSERVATION_PERIOD, DRUG_EXPOSURE). Each YAML file describes the mapping logic for a target OMOP table (Fig 2) and contains three sections: (1) name of the OMOP table being mapped (YAML field *name*), (2) definition of primary keys used by the ETL framework to manage the load (insert) operations (YAML field *primary_key)*, and (3) mapping rules for each column in the targeted OMOP table (YAML field *columns*).

### Defining primary keys

**Fig 3:**
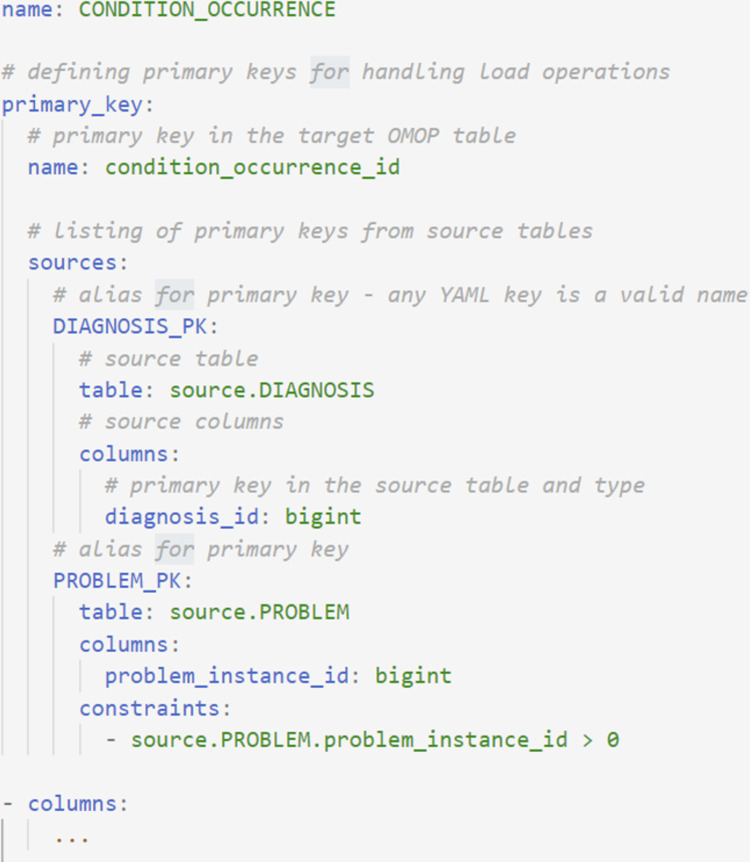
Definition of primary keys in YAML. Definition of the primary key for the OMOP CONDITION_OCCURRENCE table, which is populated—in the illustrated example—with the data from two source tables: DIAGNOSIS and PROBLEM. For the framework to handle the load operations, the primary keys of each source table must be defined under the *sources* YAML field.

The first step in the ETL framework maps every row in the OMOP table to all of the relevant rows in the source table(s). The primary_key YAML field (Figs 2 and 3) defines how to construct the primary key of the OMOP table and whether it is composed of one or more sources. The framework uses definitions of primary keys to handle the load operations. In cases where an OMOP table is populated with the data from a single source table (as in the case of populating the OMOP Person table with data from the CERNER Person table, Fig 2), the framework directly derives the primary key for the OMOP table from the primary key in the source table. When the primary key of the source table does not have a compatible type for the primary key of the OMOP table, such as when the source table has a composite primary key, the ETL framework creates an intermediate table that maps the rows from the source to the target table. Similarly, in cases where the data from multiple source tables are used to populate a single OMOP table, i.e. the OMOP table represents a union of two or more source tables (Fig 3), multiple primary key sources can be defined. Here the ETL framework creates an intermediate table with all the relevant information (the source unique identifier) for each source table and maps each row from the source tables to the appropriate row in the target table.

Fig 3 illustrates the primary key definition for the CONDITION_OCCURRENCE OMOP table, which is populated by mapping the medical codes from two CERNER tables: (1) diagnosis and (2) problem. The primary key YAML field defines a source for each of the diagnosis and problem tables, along with an alias ("DIAGNOSIS_PK" and "PROBLEM_PK”) used to distinguish between each source table when writing mapping logic (Fig 5).

### Defining columns

**Fig 4:**
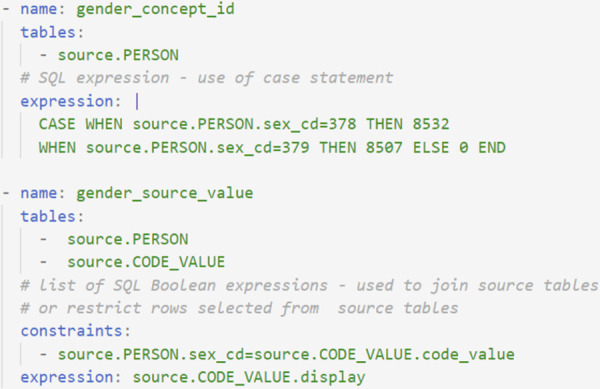
Use of PostgreSQL syntax to define mapping logic. An example of mapping logic to populate the gender_concept_id and the gender_source_value columns in the OMOP PERSON table. The transformation to populate gender_concept_id uses a CASE statement (if-then-else). The transformation to populate gender_source_value uses the data from two source tables, with the constraint indicating how these two source tables are joined.

The information needed to define the extract and transform operations from source data to an OMOP column are: (1) the name of the targeted OMOP column (YAML field *name*), (2) a listing of one or more source tables containing the data needed to populate the target field (YAML field *tables*), and (3) an SQL expression defining how one or more fields from the source table(s) map to the OMOP field (YAML field *expression*). Fig 2 shows the extract and transform operations to populate two columns (year_of_birth and death_datetime) of the PERSON OMOP table. The *expression* field supports PostgreSQL syntax, enabling the use of all functions and syntax supported by PostgreSQL (see Fig 4). For complex mapping logic, the *tables* field also supports SQL select queries.

When data from multiple source tables is needed to populate an OMOP field, as illustrated in Fig 4, the *constraints* field (a Boolean PostgreSQL expression) can be used to define how the source tables are joined and to filter rows from the source tables that meet the conditions listed. Constraints can also be used with a single source table and to restrict fields on the basis of primary keys (as shown in Fig 3). See S1 File for an example of the SQL generated for Fig 3.

### Multiple rules per column

**Fig 5:**
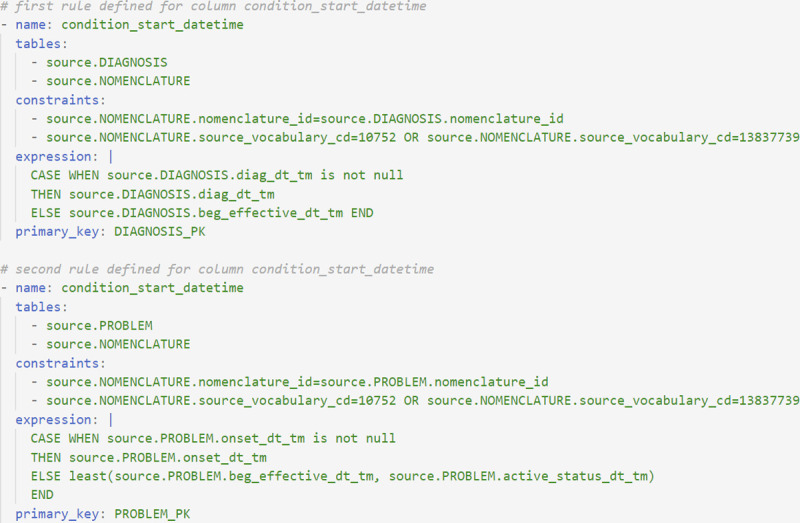
Example of complex mapping rules. Two rules are used to map diagnosis records and problem records from multiple source tables to the condition_start_date field in the OMOP CONDITION_OCCURRENCE table. Multiple rules can be written to map source data to a single OMOP column, dividing complex logic into queries that are easier to read and debug.

The mapping logic defines operations on a column-by-column basis, but in complex cases, all the rows needing to be mapped may be updated using separate rules. This is especially useful for breaking down complex logic into rules that map subsets of a single column. Fig 5 shows an example of two rules mapping source data to the condition_start_date OMOP column in the CONDITION_OCCURRENCE OMOP table (https://github.com/OHDSI/TheBookOfOhdsi). This table contains records indicating the presence of a disease or medical condition. In Fig 5, one rule is used to map diagnosis events, and the second rule maps problem events to OMOP condition occurrence events. The mapping operations in Fig 5 will result in (1) the dates of diagnosis events from the source DIAGNOSIS table and (2) the dates of problem events from the source PROBLEM table being inserted as entries into the target CONDITION_OCCURRENCE OMOP table. When multiple primary keys are defined, the alias of the primary key is used to indicate the primary key corresponding to a particular rule.

### Reducing repetition in mapping logic

**Fig 6:**
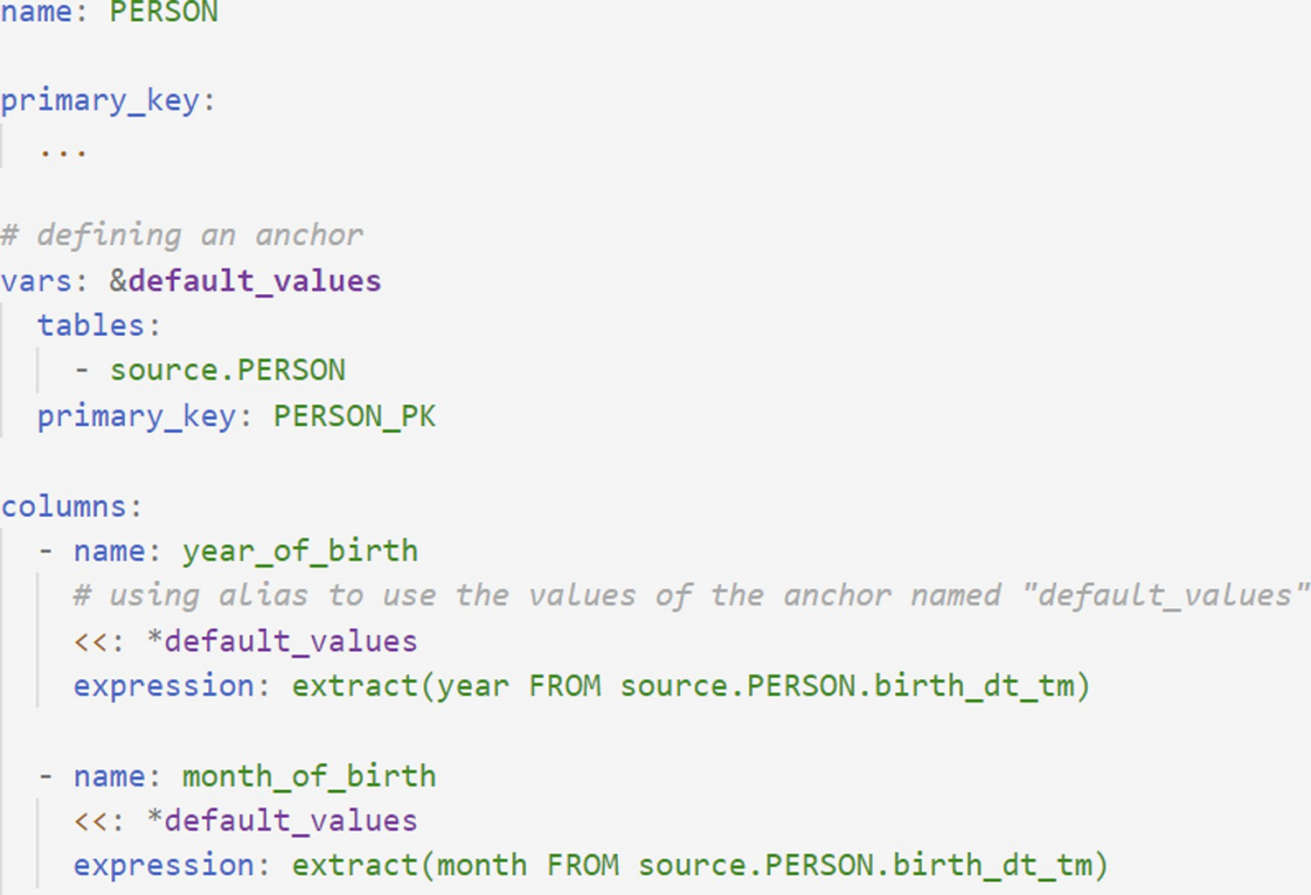
Use of YAML anchors and aliases. Rules for mapping from the CERNER PERSON table to the OMOP PERSON table, using YAML anchors to define a set of default values to be used by the column fields.

The use of YAML allows mapping rules to be written using YAML anchors and aliases. Anchors and aliases enable YAML fields to be defined once and reused multiple times, removing repetition in the YAML files. Fig 6 shows an example of an anchor being defined (“default_values”), which is subsequently used in the two columns year_of_birth and death_datetime.

### Web application

A web application (http://www.omop.link/) provides end-users with API access to the YAML to-SQL compiler, including an editor for writing mapping logic, validating the DML, and default pre-filled OMOP v6 YAML for OMOP tables (see S1 Fig for web editor view). The web application converts the YAML ETL operations to an ETL SQL script containing all the executable operations to extract, transform, and load the data from the source database to OMOP. No information about the source database schema is needed, as all the necessary logic is contained in the YAML content. The use of our DML and our web application thus has the potential to support the mapping from any source SQL database to OMOP. The web application uses React, Bootstrap, and the Monaco Editor for the front-end. The backend uses the Python FastAPI library.

## Results

### System testing

The ETL framework was system tested with 16 test cases (S1 Table) consisting of different combinations of features of the DML (for example, cases where the target column is a foreign key or the target table involves primary keys from multiple source tables). The selection of the most common features was guided by our mapping of CERNER electronic health records used by Australian Local Health Districts to OMOP CDM. Once these test cases were identified, we chose columns in the OMOP CDM tables that were appropriate candidates for each test case. We then manually generated synthetic CERNER source data and the expected mapped OMOP data for the 16 test cases for comparison against the output of the ETL framework (see S2 Fig for a diagram of how the CERNER source test data was associated with OMOP tables). Finally, we wrote the corresponding YAML files using our DML and generated an SQL script using our compiler. The test CERNER data was converted to OMOP by executing the SQL script against a PostgreSQL database. The resulting OMOP data was then compared with the manually mapped data, with the resulting OMOP data looking as expected by the OMOP CDM.

Throughout the development lifecycle, the system was also unit tested. The unit tests are included as part of the open-source software available in https://github.com/clinical-ai/omop-etl.

### Readability

Code Listing 1 in S2 File shows a single SQL statement that maps from the MIMIC-III database—comprised of ICU patients from a large tertiary care hospital—to the OMOP PERSON table [20, 21]. Code listing 1 is an example of a spaghetti query, a complex monolithic SQL query. Reading the spaghetti query from Code listing 1 involves understanding all the elements in the query: three tables, two left joins, three sub-queries, 18 source fields, and 18 target fields. The WITH clause and the structure of the INSERT statement create two degrees of separation between source and target fields, affecting readability. Even for one-to-one mappings (i.e. year_of_birth, gender_source_value), the 18 variables in the insert statement must be mentally lined up with the 18 variables in the SELECT statement to determine which variable is which.

Code listing 2 in S3 File shows the equivalent mapping from the MIMIC-III database to the OMOP PERSON table using our framework, which breaks down the mapping of the entire table into smaller tasks: mapping column by column. The structure imposed by YAML increases the number of lines compared to Code listing 1 but lowers the time and complexity involved in reading the spaghetti query. The source fields used to populate the target fields are placed within the same YAML block, removing the separation in the spaghetti query from Code listing 1. The structured blocks in YAML are also easier to understand for non-experts compared to the single SQL statement, because the YAML keys use names that are self-explanatory: columns, tables, expression, constraints.

### Development and version control

The spaghetti query from Code listing 1 (S2 File) is not amenable to multiple developers working on it at the same time, due to all the interconnected parts in the query (the sub-queries and the joins). In contrast, development with our YAML framework enables developers to work concurrently on the mapping of different columns of a table. This also integrates effectively with source control, with changes reflected with the addition or deletion of YAML blocks, as opposed to changes in the middle of a spaghetti query.

### Refactoring and maintainability

The spaghetti query from Code listing 1 (S2 File) has a higher refactoring and maintenance cost than the equivalent logic written using our ETL framework. These costs are a result of the poor readability of spaghetti queries. Refactoring and maintenance costs are even higher if a developer other than the original developer does the refactoring or maintenance, a common occurrence in the lifecycle of software projects, because the new developer must understand the code from scratch. Any schema change to the source data or the target data will incur the cost of understanding the spaghetti query (to determine where to make changes) and introduce the risk of compromising the integrity of the query (as any change has the potential to affect all fields).

The YAML equivalent in Code listing 2 (S3 File) lowers the cost of refactoring because only the relevant YAML blocks need to be understood and changed, by the original or new developers. The risk of introducing errors is minimized because any new changes to the mapping of a column does not affect any other column. Any schema changes requiring new table joins, are easier to write because there is no dependency on other columns. For example, adding or editing a left join to a column using our YAML framework involves the simple task of adding or modifying a single line under the constraints key. This can be achieved easily and independently from the other columns, as well as from any other constraint within the column under consideration.

## Discussion

Our work was driven by the need for tools that support ETL processes when mapping health datasets to OMOP. This new ETL design approach is driven by the design principles of column-by-column mapping of data to OMOP, maximizing readability, and standardizing the writing of ETL operations for mapping datasets to OMOP. Our framework divides the ETL process into (1) a core ETL pipeline that reads and executes extract-transform operations from YAML files, and (2) YAML files that organize SQL snippets defining ETL operations on a column-by-column basis. This approach makes the core ETL pipeline reusable to other research groups, accessible via a web application, with users being responsible for writing the YAML files with the ETL operations. It also enables our tool to be used with sensitive health datasets, as our web application only relies on the YAML content to generate the ETL script that can be used in various deployment and secure environments. The definition of a YAML schema in our DML also provides validation of YAML files, ensuring that rules written by users with our DML follow the right structure, standardizing the writing of OMOP mapping rules.

In contrast to an ETL process written entirely in a programming language such as Python (or otherwise), our approach stores the ETL operations in separate YAML files, making them easily accessible and promoting transparency of the ETL process. In contrast to an ETL process written entirely as an SQL script, the structure of the YAML configuration leads to readable ETL logic by defining operations on a column-by-column basis. Our proposed framework provides an alternative to existing ETL pipelines and can be used in combination with existing ETL frameworks, expanding the toolbox for mapping complex datasets to OMOP.

The column-by-column processing of our framework tackles the spaghetti query anti-pattern [22], as forcing users to think of data manipulation on a column-by-column basis is a form of divide-and-conquer that encourages users to write simple queries. This is in contrast to mapping data on a row-by-row basis, with a single complex (spaghetti) query defining the transformations for all the columns of a table. Writing rules on a column-by-column basis also allows for mapping logic to be written for a subset of OMOP, with later additions simply consisting of adding column mappings to the YAML files. Hence, logic added to map new columns does not affect or change prior column definitions, making it less likely that bugs will be introduced as a result of revising mapping logic. Finally, the column-centric approach also allows flexibility in the mapping process, afforded by both the SQL and YAML syntax, enabling the same logic to be written in different ways.

The resulting ETL operations are easier to read and understand in comparison to spaghetti queries. The readability facilitates debugging, refactoring, and understanding by users other than the original writer of the mapping logic. This serves to minimize the technical debt associated with maintaining the mapping logic and “bad smells” (code suggestive of design problems) of complex SQL queries [23, 24]. The readability of our DML also enables the sharing of the mapping logic with stakeholders who are not database experts and across institutions mapping data to OMOP. Sharing mapping logic, or subsets thereof, is as simple as sharing the YAML files.

Our web application and ETL framework have several advantages for health datasets and sensitive data. The web application does not need access to the source data. Users have the freedom to write rules inside a secure environment or within firewalls meant to protect sensitive health data. Because rules do not contain sensitive information, they can be uploaded to the web application, converted into an ETL SQL script, which can then be executed in any number of environments.

An OMOP CDM goal is to increase the transparency and replicability of real-world evidence research. If the first step in performing these research studies—the mapping of the source data to OMOP—is not transparent, reproducibility may be compromised. Our ETL framework and DML prioritize mapping logic standardization and readability, promoting transparency and reproducibility.

### Comparison with existing literature

Rabbit in a Hat is a graphical documentation tool for generating mapping requirements [19], with mappings defined by drawing arrows from source tables to the corresponding columns in OMOP tables. When mapping a large number of tables with a large number of columns, confusing visual clutter is a disadvantage, and it does not lend itself to complex mapping logic (multiple source tables used to populate one OMOP column). Rabbit in a Hat generates a mapping Word document (a requirements specification), that must then be coded by software engineers or a database expert. It can also generate an SQL skeleton including all fields to be mapped, saving developers the time to copy field names to SQL, but it still requires the actual logic to be implemented.

Our DML and YAML syntax can easily be integrated in the notes section of Rabbit in a Hat, providing users with a structured format to define the transformation logic associated with the arrows drawn between source and target fields. The readability and YAML structure also enable stakeholders from medical institutions to complete some of the YAML fields in Rabbit in a Hat, saving time for the developer who finalizes the mapping logic. The DML and YAML syntax can also be incorporated into the notes section of any graphical ETL tool, such as Talend Open Studio.

### Limitations

For complex health databases, such as CERNER EHR, the most challenging aspect of mapping data to OMOP is determining the logic that will best fit the source data to the OMOP CDM constraints with minimal data loss [15]. Our ETL framework does not address this need, and instead focuses on the standardization of mapping logic and improving transparency of the ETL process. However, the research community and health institutions need more tools to support this process, such as OHDSI’s White Rabbit.

Vocabulary mapping is a particularly challenging aspect that is commonly highlighted in studies mapping to OMOP or standardizing medical terminologies [9, 25]. As such, improvements to tools such as Usagi [26] and natural language processing approaches are needed to support vocabulary mapping. This effort has been left for future work.

The Web API tool currently generates an ETL PostgreSQL script. However, this can be easily extended to generate scripts for other relational database management systems (MySQL, Oracle) and in more dynamic scripting languages (Python, R) as future work.

Our ETL framework has undergone unit and system testing, but no user study or survey has been conducted to quantify the benefits of our approach. The discussion of benefits of our approach is based on established good software engineering practice and hands-on experience using the framework to map CERNER data to OMOP.

## Conclusion

The ETL framework proposed in this study was developed as part of our effort to map the electronic health records of local health districts in Australia to OMOP. Our team is developing new tools to improve the current mapping efforts to OMOP, enabling institutions to map datasets to OMOP at a lower cost and complexity, helping to build capacity for health services to partner in advancing data science. The design of the ETL framework is also driven by the goals of the OHDSI community, to increase transparency and reproducibility of research, and the sharing of tools that will facilitate cross-institutional research. Our ETL framework achieves this through a DML that is readable and easy to share, and a web application enabling research teams to use our DML for mapping their data to OMOP.

## Supporting information

S1 File SQL script generated by the framework. The YAML used to create this SQL script is illustrated Fig 2 of the manuscript. This script maps data to the year_of_birth and death_datetime of the OMOP Person table. The mapping table maps the rows in the target table (OMOP Person) to the source tables, in this case being the CERNER Person table.
(PDF)

S2 File Code listing 1. SQL script used to map data from the MIMIC-III database to the OMOP PERSON table.
(PDF)

S3 File Code listing 2. YAML script used to map data from the MIMIC-III database to the OMOP PERSON table. Equivalent to the SQL script from Code Listing 1.
(PDF)

S1 Fig ETL framework web application. The web application allows users to enter their YAML mapping logic, which gets converted to an ETL SQL script that can be executed in a deployed environment. This is accessible at https://www.omop.link.
(PNG)

S2 Fig Mapping of simulated CERNER data to OMOP. This mapping captures the data conversion to test features of the ETL framework.
(PNG)

S1 Table Validation test cases. Test cases to validate the ETL framework, with the collective set of test cases evaluating all features of the ETL framework.
(PDF)
