# Supplementary material for: Extract, transform, load framework for the conversion of health databases to OMOP
Source: PLoS One. 2022 Apr 11;17(4):e0266911. doi: 10.1371/journal.pone.0266911 (PMC9000122; doi:10.1371/journal.pone.0266911)
Supplement: S1 File — The YAML used to create this SQL script is illustrated Fig 2 of the manuscript. This script maps data to the year_of_birth and death_datetime of the OMOP Person table. The mapping table maps the rows in the target table (OMOP Person) to the source tables, in this case being the CERNER Person table. (PDF) [file pone.0266911.s001.pdf]

S1 File

```
create table mapping.PERSON (id serial PRIMARY KEY, PERSON_person_id bigint null);

insert into
  mapping.PERSON (PERSON_person_id)
select
  PERSON.person_id as PERSON_person_id
from
  source.PERSON;

insert into
  omop.PERSON (person_id)
select
  mapping.PERSON.id
from
  mapping.PERSON;

update
  omop.PERSON
set
  year_of_birth = extract(
    year
    FROM
      source.PERSON.birth_dt_tm
  )
from
  mapping.PERSON,
  source.PERSON
where
  (omop.PERSON.person_id = mapping.PERSON.id)
  and (
    source.PERSON.person_id = mapping.PERSON.PERSON_person_id
  );

update
  omop.PERSON
set
  death_datetime = deceased_dt_tm
from
  mapping.PERSON,
  source.PERSON
where
  (omop.PERSON.person_id = mapping.PERSON.id)
  and (
    source.PERSON.person_id = mapping.PERSON.PERSON_person_id
  );
```
