# Supplementary material for: Extract, transform, load framework for the conversion of health databases to OMOP
Source: PLoS One. 2022 Apr 11;17(4):e0266911. doi: 10.1371/journal.pone.0266911 (PMC9000122; doi:10.1371/journal.pone.0266911)
Supplement: S2 File — SQL script used to map data from the MIMIC-III database to the OMOP PERSON table. (PDF) [file pone.0266911.s002.pdf]

S2 File

```
WITH
"patients" AS (SELECT subject_id, mimic_id as person_id, CASE WHEN gender ='F' THEN 8532 WHEN GENDER = 'M' THEN 8507 ELSE NULL END as gender_concept_id, extract(year FROM dob) as year_of_birth, extract(month FROM dob) as month_of_birth, extract(day FROM dob) as day_of_birth, dob as birth_datetime, gender as gender_source_value FROM patients),
"gcpt_ethnicity_to_concept" AS (SELECT ethnicity, race_concept_id as race_concept_id, ethnicity_concept_id as ethnicity_concept_id FROM gcpt_ethnicity_to_concept),
"admissions" AS (SELECT DISTINCT ON (subject_id) subject_id, first_value(ethnicity) OVER(PARTITION BY subject_id ORDER BY admittime ASC) as race_source_value FROM admissions)
 INSERT INTO :OMOP_SCHEMA.PERSON
 (
     person_id
   , gender_concept_id
   , year_of_birth
   , month_of_birth
   , day_of_birth
   , birth_datetime
   , race_concept_id
   , ethnicity_concept_id
   , location_id
   , provider_id
   , care_site_id
   , person_source_value
   , gender_source_value
   , gender_source_concept_id
   , race_source_value
   , race_source_concept_id
   , ethnicity_source_value
   , ethnicity_source_concept_id
 )
 SELECT
  patients.person_id
, patients.gender_concept_id
, patients.year_of_birth
, patients.month_of_birth
, patients.day_of_birth
, patients.birth_datetime
, gcpt_ethnicity_to_concept.race_concept_id
, 0 as ethnicity_concept_id
, null::integer location_id
, null::integer provider_id
, null::integer care_site_id
, patients.subject_id::text person_source_value
, patients.gender_source_value
, null::integer gender_source_concept_id
, admissions.race_source_value
, null::integer race_source_concept_id
, null::text ethnicity_source_value
, null::integer ethnicity_source_concept_id
FROM patients
LEFT JOIN admissions USING (subject_id)
LEFT JOIN gcpt_ethnicity_to_concept ON (admissions.race_source_value = gcpt_ethnicity_to_concept.ethnicity);
```
