# Supplementary material for: Extract, transform, load framework for the conversion of health databases to OMOP
Source: PLoS One. 2022 Apr 11;17(4):e0266911. doi: 10.1371/journal.pone.0266911 (PMC9000122; doi:10.1371/journal.pone.0266911)
Supplement: S3 File — YAML script used to map data from the MIMIC-III database to the OMOP PERSON table. Equivalent to the SQL script from Code Listing 1. (PDF) [file pone.0266911.s003.pdf]

S3 File

```yaml
name: PERSON
primary_key:
  name: person_id
  sources:
    PERSON_PK:
      table: patients
      columns:
        person_id: bigint

ethnicity_tables: &ethnicity_tables
  tables:
      - patients
      - alias: ADMISSIONS_QUERY
        query: |
          SELECT DISTINCT ON (subject_id) subject_id, first_value(ethnicity)
          OVER(PARTITION BY subject_id ORDER BY admittime ASC) as race_source_value
          FROM admissions
  constraints:
    - patients.subject_id = ADMISSIONS_QUERY.subject_id
    - ADMISSIONS_QUERY.race_source_value = gcpt_ethnicity_to_concept.race_source_value

columns:
  - name: gender_concept_id
    tables:
      - patients
    expression: |
      CASE
          WHEN patients.gender = 'F' THEN 8532
          WHEN patients.gender = 'M' THEN 8507
          ELSE 0
      END

  - name: year_of_birth
    tables:
      - patients
    expression: extract(year from patients.dob)

  - name: month_of_birth
    tables:
      - patients
    expression: extract(month from patients.dob)

  - name: day_of_birth
    tables:
      - patients
    expression: extract(day from patients.dob)

  - name: birth_datetime
    tables:
      - patients
    expression: patients.dob

```

```yaml
  - name: race_concept_id
    *ethnicity_tables
    expression: gcpt_ethnicity_to_concept.race_concept_id

  - name: ethnicity_concept_id
    *ethnicity_tables
    expression: gcpt_ethnicity_to_concept.ethnicity_concept_id

  - name: person_source_value
    tables:
      - patients
    expression: CAST(patients.subject_id AS STRING)

  - name: gender_source_value
    tables:
      - patients
    expression: patients.gender_source_value

  - name: race_source_value
    *ethnicity_tables
    expression: admissions.race_source_value
```
