# Supplementary figures and images for: Extract, transform, load framework for the conversion of health databases to OMOP
Source: PLoS One. 2022 Apr 11;17(4):e0266911. doi: 10.1371/journal.pone.0266911 (PMC9000122; doi:10.1371/journal.pone.0266911)

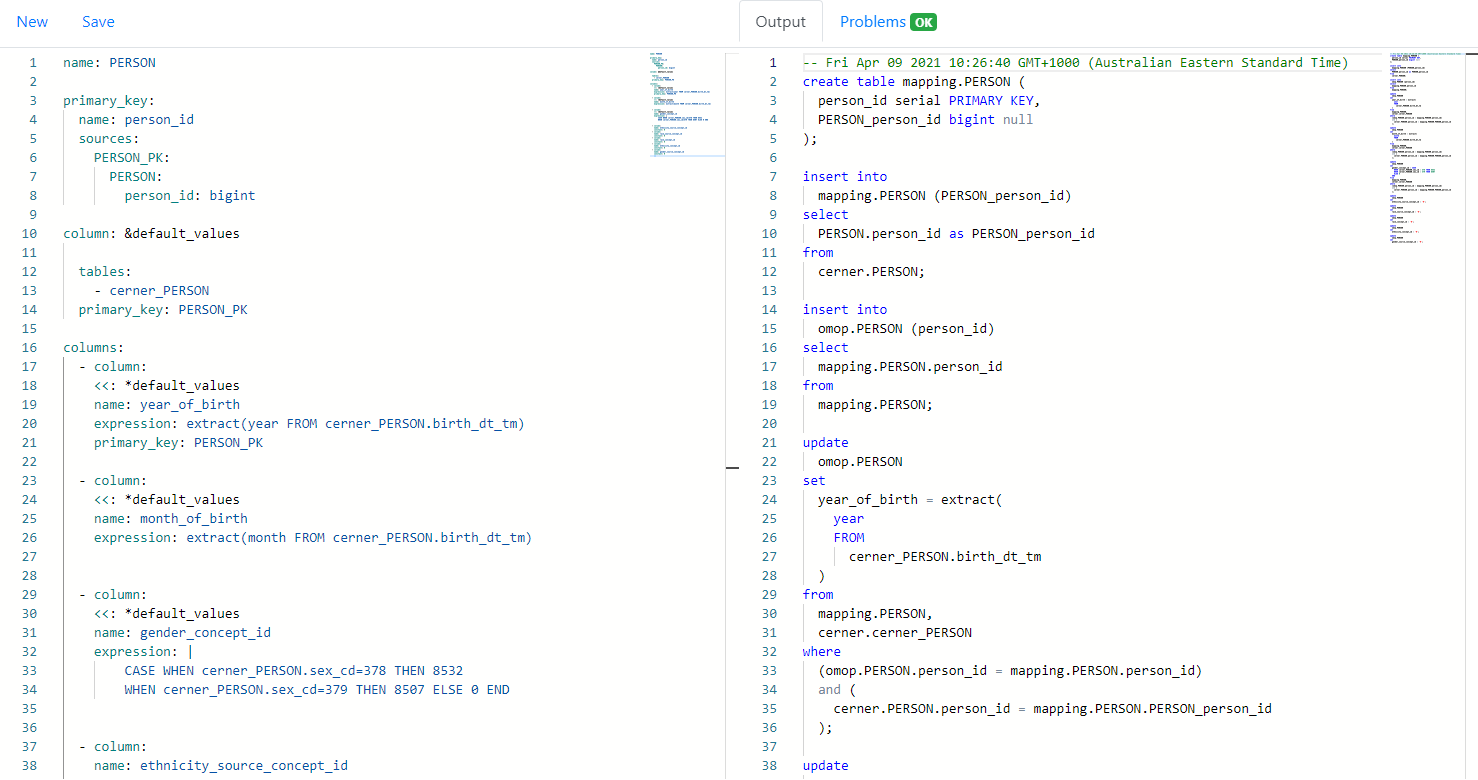

Supplement: S1 Fig — The web application allows users to enter their YAML mapping logic, which gets converted to an ETL SQL script that can be executed in a deployed environment. This is accessible at https://www.omop.link. (PNG) [file pone.0266911.s004.png]

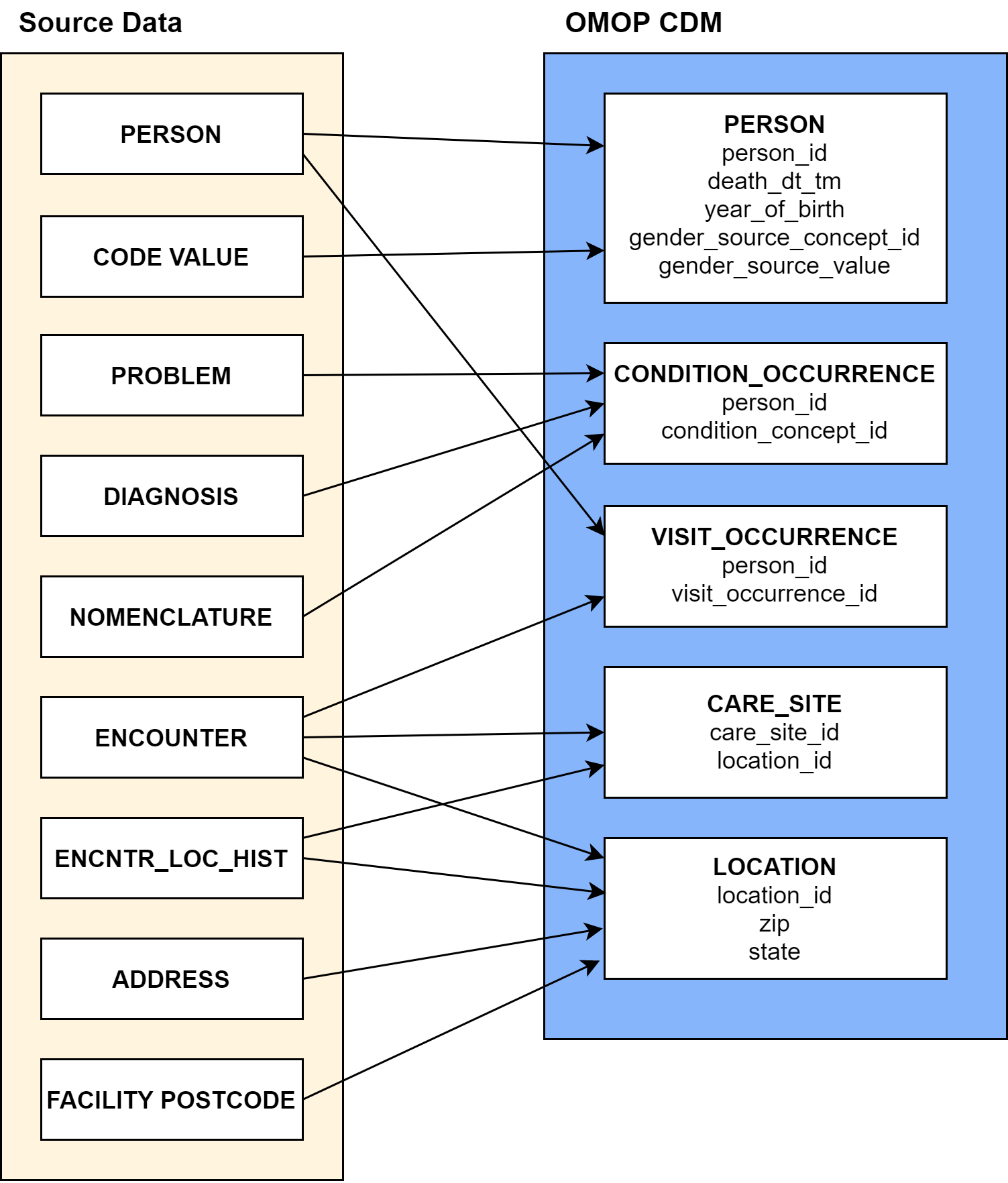

Supplement: S2 Fig — This mapping captures the data conversion to test features of the ETL framework. (PNG) [file pone.0266911.s005.png]
