# Supplementary material for: Extract, transform, load framework for the conversion of health databases to OMOP
Source: PLoS One. 2022 Apr 11;17(4):e0266911. doi: 10.1371/journal.pone.0266911 (PMC9000122; doi:10.1371/journal.pone.0266911)
Supplement: S1 Table — Test cases to validate the ETL framework, with the collective set of test cases evaluating all features of the ETL framework. (PDF) [file pone.0266911.s006.pdf]

S1 Table

Test case	Primary keys from multiple source tables	Column is Primary Key	Column is Foreign Key	Expression	Column involves constraints	OMOP Target Column
1		✓		Simple	✓	PERSON person_id
2				Simple		PERSON death_dt_tm
3				Complex		PERSON year_of_birth
4				Constant		PERSON gender_source_concept_id
5				Simple	✓	PERSON gender_source_value
6		✓		Simple	✓	VISIT_OCCURRENCE visit_occurrence_id
7			✓	Simple	✓	VISIT_OCCURRENCE person_id
8	✓	✓		Simple	✓	CONDITION_OCCURRENCE condition_occurrence_id
9	✓	✓		Simple	✓	LOCATION location_id
10	✓			Simple	✓	CONDITION_OCCURRENCE condition_concept_id
11	✓			Complex		LOCATION zip
12	✓			Constant		LOCATION state
13	✓			Simple	✓	CONDITION_OCCURRENCE condition_concept_id
14	✓		✓	Simple	✓	CONDITION_OCCURRENCE person_id
15	✓	✓		Simple	✓	CARE_SITE care_site_id
16	✓		✓	Simple	✓	CARE_SITE location_id
